# Potential role for pyruvate kinase M2 in the regulation of murine cardiac glycolytic flux during *in vivo* chronic hypoxia

**DOI:** 10.1042/BSR20203170

**Published:** 2021-06-02

**Authors:** Michal K. Handzlik, David J. Tooth, Dumitru Constantin-Teodosiu, Paul L. Greenhaff, Mark A. Cole

**Affiliations:** 1 School of Life Sciences, University of Nottingham Medical School, Queen's Medical Centre, Nottingham, U.K.; 2 MRC Versus Arthritis Centre for Musculoskeletal Ageing Research, School of Life Sciences, Queen’s Medical Centre, University of Nottingham, Nottingham, U.K.

**Keywords:** hypoxia, myocardium, pyruvate kinase M2

## Abstract

Carbohydrate metabolism in heart failure shares similarities to that following hypoxic exposure, and is thought to maintain energy homoeostasis in the face of reduced O_2_ availability. As part of these *in vivo* adaptations during sustained hypoxia, the heart up-regulates and maintains a high glycolytic flux, but the underlying mechanism is still elusive. We followed the cardiac glycolytic responses to a chronic hypoxic (CH) intervention using [5-^3^H]-glucose labelling in combination with detailed and extensive enzymatic and metabolomic approaches to provide evidence of the underlying mechanism that allows heart survivability. Following 3 weeks of *in vivo* hypoxia (11% oxygen), murine hearts were isolated and perfused in a retrograde mode with function measured via an intraventricular balloon and glycolytic flux quantified using [5-^3^H]-glucose labelling. At the end of perfusion, hearts were flash-frozen and central carbon intermediates determined via liquid chromatography tandem mass spectrometry (LC-MS/MS). The maximal activity of glycolytic enzymes considered rate-limiting was assessed enzymatically, and protein abundance was determined using Western blotting. Relative to normoxic hearts, CH increased *ex vivo* cardiac glycolytic flux 1.7-fold with no effect on cardiac function. CH up-regulated cardiac pyruvate kinase (PK) flux 3.1-fold and cardiac pyruvate kinase muscle isoenzyme M2 (PKM2) protein content 1.4-fold compared with normoxic hearts. CH also augmented cardiac pentose phosphate pathway (PPP) flux, reflected by higher ribose-5-phosphate (R5P) content. These findings support an increase in the covalent (protein expression) and allosteric (flux) control of PKM2 as being central to the sustained up-regulation of the glycolytic flux in the chronically hypoxic heart.

## Introduction

The metabolic profile of carbohydrate metabolism in myocardium in heart failure shares many similarities with that during sustained hypoxic exposure [1,2]. Maintaining energy homoeostasis during hypoxia requires extensive metabolic reprogramming aimed at supporting ATP provision in the face of reduced O_2_ availability [3]. Metabolic reprogramming following short-term (24–96 h) *in vitro* hypoxia, which comprises the majority of information available in the literature, involves up-regulation of enzymes that accelerate the glycolytic flux [4–6], down-regulation of β-oxidation medium- and long-chain dehydrogenases, and electron transport chain components to suppress oxidative phosphorylation [7,8]. These events are regulated by the oxygen-sensing transcription factor hypoxia-inducible factor (HIF)-1α [8,9]. However, the adaptations identified during sustained hypoxia in the intact heart, cover a particular set of enzymes including hexokinase (HK), lactate dehydrogenase and pyruvate kinase (PK) [10–12], and are considered to be part of the process of maintaining energy homoeostasis. Therefore, it is remains to be fully established how mammalian heart maintains up-regulated cardiac glycolytic flux during prolonged *in vivo* hypoxia.

Glycolytic flux is classically considered to be regulated by four rate-limiting enzymes, HK, phosphofructokinase (PFK), glyceraldehyde-3-phosphate dehydrogenase (GAPDH) and PK [13–15]. Although HK and PFK use two molecules of ATP at the beginning of the pathway, phosphoglycerate kinase (PGK) and PK result in the formation of four ATP molecules for each molecule of glucose degraded to pyruvate [15]. In an attempt to assess the physiological importance of individual glycolytic steps, Tanner et al. [16] sequentially and transiently overexpressed in a cell line human isoenzymes catalysing every glycolytic step from glucose uptake to lactate ion export and identified glucose uptake, fructose-1,6-bisphosphate (FBP) production and lactate ion export as key glycolytic flux controlling steps. In cardiac muscle exposed to *in vivo* hypoxia, some [10,11,17], although not all [18], have demonstrated up-regulated glucose uptake capacity in conjunction with increased HK activity. So far, therefore, the mechanisms that up-regulate the glycolytic flux in cardiac muscle during hypoxia are still controversial.

PK catalyses the last step of glycolysis, i.e. the conversion of phosphoenolpyruvate (PEP) into pyruvate. Due to its broad allosteric regulation, PK has been suggested to be an important regulator of glycolytic flux in cancer, failing heart and kidney disease [19–22]. Of the four existing PK protein isoforms, each one with different kinetic properties, adult PK muscle isoenzyme 1 (PKM1) and foetal PK muscle isoenzyme 2 (PKM2) have the highest and mutually exclusive expression in multiple mammalian tissues [23]. PKM2 is predominantly expressed in embryonic cardiac and skeletal muscle and becomes progressively replaced by PKM1 during post-natal developmental, which is consistent with increased tissue oxygen availability [20]. Considering that PKM1 and PKM2 differ in 22 amino acid residues, they exhibit significantly distinct biochemical properties. Although PKM2 displays lower activity than PKM1, which is constitutively the active form [19], PKM2 is controlled by a wide variety of regulatory mechanisms including allosteric activation by amino acid serine and FBP, and inhibition by post-translational phosphorylation and acetylation [24–27]. Additional *in vitro* studies have confirmed that PKM1 and PKM2 expressions appear to be collectively subjected to multiple controlling factors, including transcriptional repression [28], alternative splicing [20] and hypoxia [22,29]. However, little is known about the mechanisms regulating cardiac PK activity and protein isoform levels during prolonged *in vivo* hypoxia.

Against the background of the above, we hypothesised that underlying mechanisms by which cardiac muscle up-regulates glycolytic flux *in vivo* during chronic hypoxia are intimately linked to the role of PK. To test our hypothesis, a comprehensive profile of metabolic adaptation to chronic hypoxia following the exposure of mice to 3 weeks of physiological hypoxia versus normoxia was acquired. This included enzymatic and wide range metabolomic profiling (glycolytic, tricarboxylic acid cycle and pentose phosphate pathway (PPP)) to fingerprint each intermediate step of glycolysis in the heart.

## Methods

### Animals

Eight-week-old male CD1 mice were purchased from a commercial breeder (Harlan, United Kingdom). All procedures were approved by and performed in accordance with the Home Office guidelines under The Animals (Scientific Procedures) Act, 1986, and the University of Nottingham guidelines.

### Chronic hypoxic housing

The effects of chronic *in vivo* hypoxia on murine cardiac function and metabolism were investigated in mice randomly assigned to either normoxic (*n*=8) or hypoxic housing (*n*=10). The mice were housed (four mice/cage) in a hypoxic chamber for 3 weeks that began with a 7-day acclimatisation period involving gradual reduction in chamber oxygen from 21 to 11% to produce graded physiological hypoxia as previously described in detail [30,31]. Chamber oxygen level was then maintained at 11% for 2–3 weeks. Chronic hypoxic (CH) housing was performed in a sealed plastic glass-fronted chamber (Medical Engineering Unit, Nottingham, U.K.). Hypoxia in the chamber was achieved by removal of O_2_ content of incoming air via a hypoxic generator (Hypoxico, U.S.A.). Control (21% O_2_) and chronically hypoxic animals were housed in the same room ensuring exposure of animals to the same temperature, humidity and 12-h light/dark cycle conditions. Standard chow diet and water were provided *ad libitum* throughout experiment. Following CH housing, all animals were exposed to room air for 1 h, to exclude effects of short-term reoxygenation on cardiac function.

### Isolated heart perfusion

All animals were anaesthetized with a terminal dose of sodium pentobarbitone (60 mg/kg of body weight i.p).Hearts were excised and arrested in ice-cold Krebs–Henseleit (KH) buffer containing (in mM) 118 NaCl, 4.7 KCl, 1.2 MgSO_4_, 2.0 CaCl_2_, 0.5 Na_2_EDTA, 11 glucose, 25 NaHCO_3_ and 1.2 KH_2_PO_4_. A blood sample was taken from the thoraic cavity to determine haemoglobin content (Haemocue AB, Ängelholm, Sweden). Hearts were then perfused in Langendorff mode under constant pressure (80 mmHg) with recirculating KH buffer containing 0.4 mM palmitate pre-bound to albumin (3%). Up to four hearts per day were perfused beginning in the morning (8.00 a.m.). Normoxic and hypoxic hearts were perfused randomly. The buffer was continually gassed with a mix of 95% O_2_ and 5% CO_2_, with the temperature maintained at 37°C. Cardiac function was measured continuously using polyethene balloon placed within the lumen of left ventricle inflated to 4–8 mmHg, determining left ventricular (LV) developed pressure (DP) and heart rate (HR). The rate pressure product (RPP) was calculated as a product of HR and DP. Following 30-min perfusion, hearts were freeze-clamped using Wollenberger clamps pre-chilled in liquid nitrogen and subsequently stored at −80°C until analysis.

### Measurements of cardiac glycolytic flux

Cardiac glycolytic flux in control and hypoxic animals was determined as previously described [30]. Briefly, 30 μCi of [5-^3^H]-glucose (PerkinElmer) was added to KH buffer before perfusion. Aliquots of recirculating buffer were collected at 5-min intervals during the perfusion protocol, and ^3^H_2_O content used to calculate glycolytic flux following Dowex anion separation (1 × 4-200, anion exchange resin, Sigma, St. Louis U.S.A.). Cardiac lactate efflux was determined spectrophotometrically using a lactate dehydrogenase coupled enzyme assay of timed buffer collections as previously described [31].

### Liquid chromatography–mass spectrometry

Frozen hearts were freeze-dried and powdered, and metabolites extracted as previously described [32]. Briefly, −20°C acetonitrile/methanol/water (40:40:20 v/v/v) solution was added to the powdered tissue and incubated at −20°C for 15 min with occasional vortexing. The sample was centrifuged at 13000 rpm for 5 min at 4°C and the supernatant collected. The residual pellet was re-extracted twice on ice, and all three supernatants were pooled. The pooled supernatants were then dried under nitrogen gas, and the pellet was resuspended in HPLC water and stored at −80°C until analysis. To determine specific compound-dependent mass spectrometry parent ion (*m/z*) and daughter ion parameters, single analyte standards, at a concentration of ∼50 μM, dissolved in 50% (v/v) acetonitrile 0.1% (v/v) NH_4_OH, were (syringe) infused at a flow rate of 5 μl/min and analysed in negative ion (ESI-) and full-scan mode (Supplementary Table S1). Triple quadrupole (Quattro Ultima; Waters) instrument parameters were optimised for precursor ions and collision energies were optimised for product (quantifier and validator) ions. Various instrumental settings were optimised to maximise the signal with the final parameters being: capillary voltage (kV) 3.2, cone 60 (arbitrary units), Hex1 40 (arbitrary units), source temperature 120°C, desolvation temperature 250°C. An autosampler (Waters 2700 Sample Manager, Waters, U.S.A.) binary gradient (Jasco PU2085) was chromatographed in reversed-phase mode using a 2.1 × 100 mm, 3.5 μm C18 column (XBrigde, Waters, U.S.A.) at room temperature with eluent A containing 10 mM tributylamine, 15 mM acetic acid and 3% (v/v) acetonitrile and eluent B being 100% acetonitrile. The mobile phase was infused at a rate of 0.2 ml/min. The gradient used was 0 min, 3% B; 0–25 min, 20% B; 25–26 min, 100% B; 26–30 min, 0% B; 30–60 min, 0% B. To minimise technical and biological variability, the samples were thawed 10–15 min before analysis and run sequentially. Retention times and transitions were analysed via multiple reaction monitoring (MRM) using MassLynx software (MassLynx, V4.0 SP4, Waters, U.S.A.). Data were quantified from the external standard curve being analysed together with samples.

### Enzyme activities

Enzymatic activities were determined in frozen heart tissues: HK, PFK, GAPDH and PK activities were determined using previously established methods [33–36]. Total and compartmental muscle lysate protein concentrations were measured using a Bradford assay, with enzyme activity expressed as U/mg of protein.

### Western blotting

An aliquot of the heart muscle was homogenised in ice-cold 50 mM Tris-HCl, pH 7.5, buffer containing 1 mM EDTA, 1 mM EGTA, 1% IGEPAL, 0.1% β-mercaptoethanol and 10 μl/ml of protease inhibitor cocktail (Sigma, St. Louis, U.S.A.). Tissue lysate was centrifuged at 13000×***g*** for 10 min at 4°C, and the supernatant was stored at −80°C. Nuclear proteins were extracted from the pellet formed following centrifugation. Specifically, the pellet was resuspended in 200 μl of 20 mM HEPES buffer, pH 8.0, containing 25% glycerol, 500 mM NaCl, 1.5 mM MgCl_2_, 0.2 mM EDTA and left on ice for 60 min. The lysate was spun at 3000×***g*** for 5 min and the supernatant collected. Homogenate protein content was determined using the bicinchoninic acid assay (Pierce, U.S.A.). Protein samples were run a 12% Bis-Tris acrylamide gel for 2 h at constant 100 V and transferred on a polyvinylidenedifluoride membrane (PVDF) for 2 h at constant 250 mA in an ice-chilled transfer tank. The membrane was blocked and incubated overnight at 4°C with rabbit anti-PKM1 antibody (1:10000, Sigma), rabbit anti-PKM2 antibody (1:1000, Cell Signaling, #D78A4), rabbit anti-CUGBP1 (1:3000, Abcam, U.K., #ab129115), rabbit anti-PDK1 (1:100000, Cell Signaling, #3820), rabbit anti-Sp3 (1:500, Santa Cruz, #sc-644) and rabbit anti-actin antibody (1:50000, Sigma, #A2066). Membranes were then washed and incubated with goat anti-rabbit HRP–conjugated secondary antibody (R&D Systems). After washing membranes were incubated with enhanced chemiluminescence (ECL) detection solution (Amersham, U.K.) and exposed to X-ray film (Kodak, U.K.).

### Statistical analysis

Distribution of the data in each group was determined using Kolmogorov–Smirnov and Shapiro–Wilk tests. Independent Student’s *t* test or Mann–Whitney tests were used to test for mean differences between groups for normally and non-normally distributed data, respectively. Two-way ANOVAs with Bonferroni’s *post-hoc* tests were used to test for mean differences between groups. Statistical significance was set at *P*≤0.05 for all analyses with the results presented as individual values and mean ± standard error of mean (SEM).

## Results

**Figure 1:**
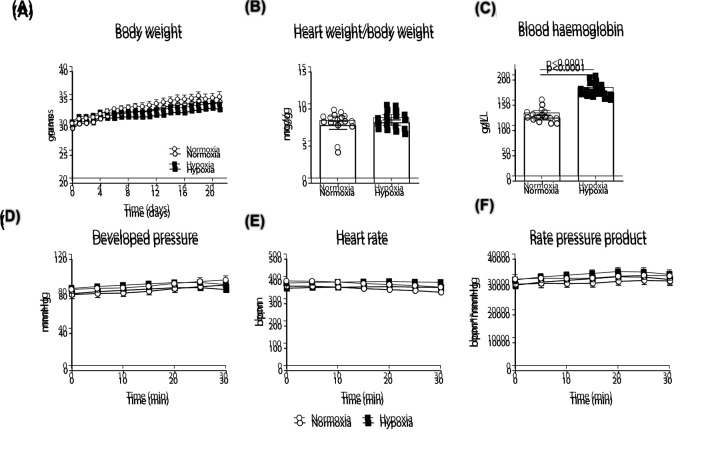
Effect of chronic *in vivo* hypoxia on cardiac morphology, blood haemoglobin levels and isolated *ex vivo* cardiac function Body weight (**A**), heart weight/body weight ratio (**B**), blood haemoglobin (**C**), LVDP (**D**), HR (**E**) and rate pressure product (**F**). Values are expressed as mean ± SEM (*n*=8–10).

There were no differences in body weight (Figure 1A) and cardiac hypertrophy between normoxic and hypoxic groups (Figure 1B), consistent with our previous work using the current CH protocol [2,30]. Sustained *in vivo* hypoxia increased blood haemoglobin in comparison to normoxic mice (Figure 1C, *P<0.0001*) without affecting the function of the perfused heart (Figure 1D–F).

**Figure 2:**
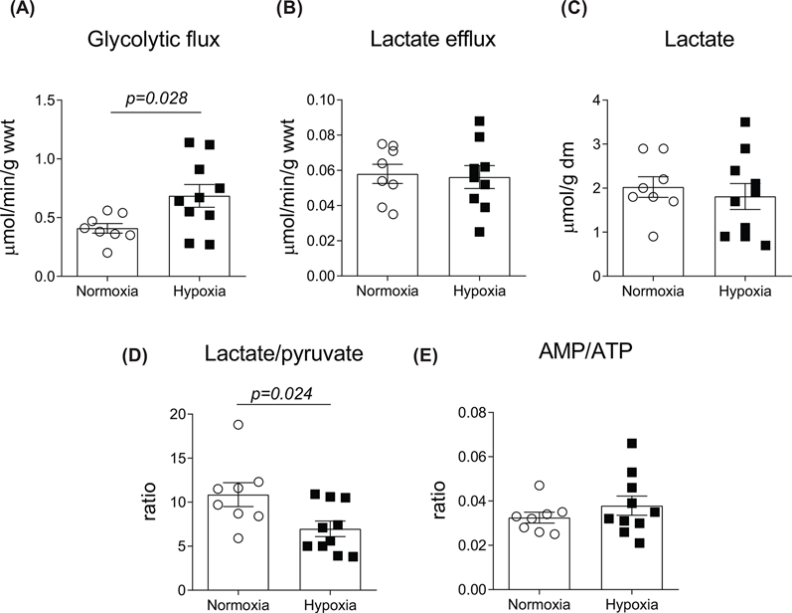
Chronic *in vivo* hypoxia increased cardiac glycolytic flux Glycolytic flux (**A**), lactate efflux (**B**), lactate content (**C**), lactate/pyruvate ratio (**D**) and AMP/ATP ratio (**E**). Values are expressed as mean ± SEM (*n*=8–10).

Next, we evaluated how sustained *in vivo* hypoxia modified cardiac glycolysis in isolated beating hearts using [5-^3^H]-glucose (Figure 2). While we found no differences in the net cardiac lactate efflux and tissue lactate content between normoxic and hypoxic hearts, chronic hypoxia resulted in 1.7-fold higher cardiac glycolytic flux relative to normoxic hearts (Figure 2A, *P=0.028*). The lactate/pyruvate ratio, an indicator of cytosolic NAD^+^/NADH ratio, was reduced in chronically hypoxic hearts relative to the normoxic group (Figure 2D, *P=0.024*).

**Figure 3:**
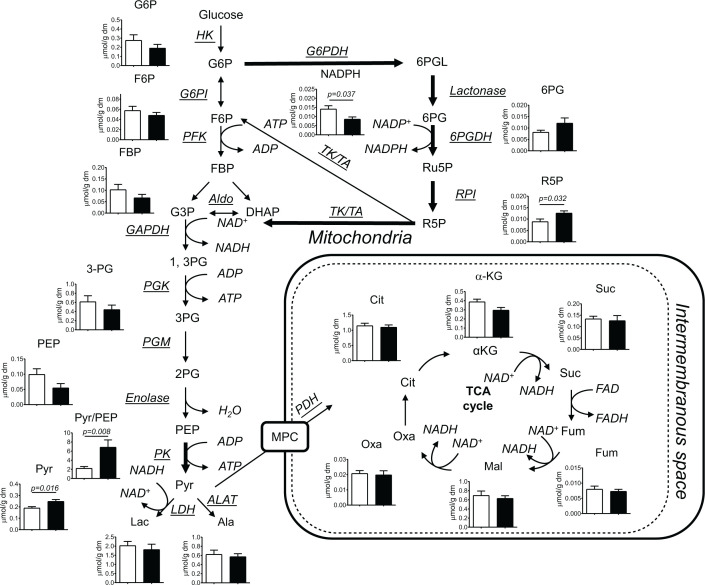
Chronic *in vivo* hypoxia altered cardiac central carbon metabolism Cardiac LC/MS/MS analysis of intermediate metabolites following chronic hypoxia. Abbreviations: ALAT, alanine aminotransferase; Aldo, aldolase; Cit, citrate; DHAP, dihydroxyacetone phosphate; F6P, fructose-6-phosphate; Fum, fumarate; G3P, glyceraldehyde-3-phosphate; G6P, glucose-6-phosphate; G6PDH, glucose-6-phosphate dehydrogenase; G6PI, glucose-6-phosphate isomerase; LDH, lactate dehydrogenase; Mal, malate; MPC, mitochondrial pyruvate carrier; Oxa, oxaloacetate; PDC, pyruvate dehydrogenase complex; PGM, phosphoglyceromutase; Pyr, pyruvate; RPI, ribose phosphate isomerase; Ru5P, ribulose-5-phosphate; Suc, succinate; TA, transaldolase; TK, transketolase; αKG, α-ketoglutarate; 1,3PG, 1,3-phosphoglycerate; 2PG, 2-phosphoglycerate; 3PG; 3-phosphoglycerate; 6PG, 6-phosphogluconate; 6PGDH, 6-phosphogluconate dehydrogenase; 6PGL, 6-phosphogluconolactone. Normoxia: open bars, Hypoxia: filled bars. Values are expressed as mean ± SEM (*n*=6–10).

To explore how chronic *in vivo* hypoxia modulated the cardiac central carbon intermediate metabolism, we utilised tandem mass spectrometry (liquid chromatography tandem mass spectrometry (LC-MS/MS)) to detect and quantify glycolytic, TCA cycle and PPP metabolites (Figure 3). LC-MS/MS analysis showed that pyruvate content in chronically hypoxic hearts was 31% higher than in the normoxic group (*P=0.016*). Additionally, pyruvate/PEP ratio, a validated marker of PK activity [37], in the hypoxic hearts was 3.1-fold higher than in normoxic hearts (*P=0.008*). Levels of PPP ribose-5-phosphate (R5P) in chronically hypoxic hearts were 42% higher than in normoxic groups (*P=0.032*). Additionally, NADPH levels in hypoxic hearts were 36% lower than in normoxic group (*P=0.037*).

**Figure 4:**
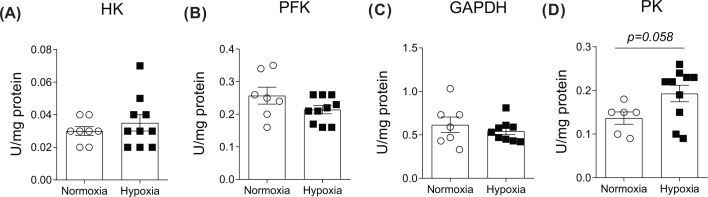
Effects of chronic hypoxia on isolated heart enzyme activity Myocardial activity of HK (**A**), PFK (**B**), GAPDH (**C**) and PK (**D**). Values are expressed as mean ± SEM (*n*=6–10).

To investigate the mechanistic basis for increased glycolytic flux in chronically hypoxic hearts, enzymatic activities of four key rate-limiting enzymes were determined (Figure 4). There were no differences for cardiac HK, PFK and GAPDH activities between normoxic and hypoxic hearts (Figure 4A–C). Cardiac PK activity in chronically hypoxic hearts tended to be higher than in normoxic group (Figure 4D, 39%, *P=0.058*).

**Figure 5:**
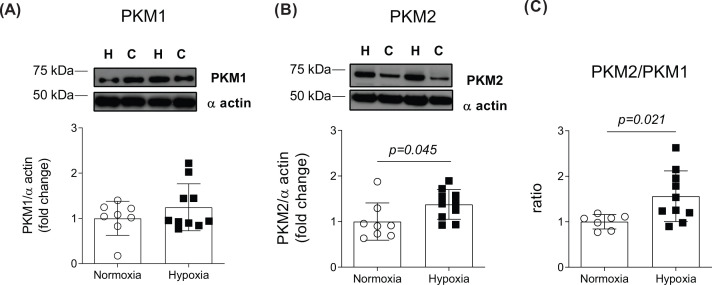
Sustained *in vivo* hypoxia up-regulated cardiac PKM2 expression Western blot analysis of PKM1 (**A**), PKM2 (**B**) and the ratio of PKM2 to PKM1 (**C**). C, control; H, hypoxia. Values are expressed as mean ± SEM (*n*=7–10).

Next, we determined if increased cardiac PK activity could be accounted for by differential PK protein isoform expression (Figure 5). As shown by Western blot analysis, no differences between normoxic and hypoxic hearts were found for PKM1 protein expression (Figure 5A). In contrast, PKM2 protein levels in the chronically hypoxic hearts were significantly greater than in normoxic hearts (1.4-fold; Figure 5B, *P=0.027*). Furthermore, the PKM2/PKM1 ratio in chronically hypoxic hearts was significantly greater compared with normoxic hearts (1.6-folds; Figure 5C, *P=0.021*).

**Figure 6:**
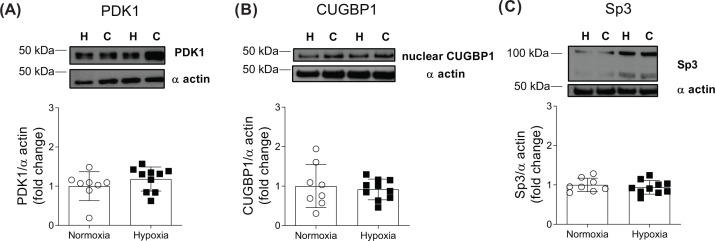
Potential upstream regulators of PKM2 protein were unaltered Western blot analysis of cardiac pyruvate dehydrogenase kinase [PDK1 (**A**)], CUG binding protein 1 [CUGBP1 (**B**)] and Sp3 (**C**) proteins following CH exposure. C, control; H, hypoxia. Values are expressed as mean ± SEM (*n*=8–10).

To further explore how chronic *in vivo* hypoxia might increase PKM2 protein levels, we measured its three different potential upstream regulators, PDK1 (HIF-1α signalling), alternative splicing (CUGBP1) and transcriptional repressor (Sp3) (Figure 6). There were no differences between normoxic and hypoxic hearts for any of measured proteins.

## Discussion

### Sustained *in vivo* hypoxia increased cardiac PKM2 protein content and flux

Here, we demonstrate that *in vivo* extended hypoxia selectively increases the PK enzymatic activity and flux, as well as the pyruvate/PEP ratio. This indicates that the metabolic responses to chronic hypoxia in mammalian heart appear to be related to the adaptive responses of this glycolytic enzyme.

Previous *in vitro* studies exploring how hypoxia modulates glycolytic capacity have provided conflicting findings. For instance, activities of all glycolytic enzymes in L8 muscle cells, except for HK, were increased following 96-h *in vitro* hypoxia (4–10% O_2_) [4]. Examining the effects of 4-week hypoxia on glycolytic enzyme activity, Martinez et al. reported increased HK, triosephosphate isomerase (TPI) and PK activities in killifish heart [38]. Furthermore, following a 28-day hypoxic exposure, activities of five glycolytic enzymes (HK, Aldolase, GAPDH, PGK and PK) were increased in guinea pig left ventricle [12], suggesting interspecies and or temporal differences in hypoxia-induced modification of glycolysis. Enolase activity, the enzymatic step used in the present study to assess glycolytic rate, is not seen to be elevated in myocardium [12]. Whilst the pattern of enzyme up-regulation on exposure to hypoxia does vary between studies, a common finding is that PK activity is increased, and warranted further investigation in the current study.

Alternative splicing of exons 9 and 10 of the *PKM* gene generates two PKM1 or PKM2 isoforms, respectively [20]. Relative to PKM1, PKM2 is a less-active isoform, although it exhibits complex regulatory circuits and exists in a dynamic dimer–tetramer form [24,27,37,40]. LC-MS/MS analysis in the present study revealed that tissue FBP concentration was not different between groups, indicating that FBP-induced allosteric activation was unlikely to account for increased cardiac PK flux. In contrast, analysis of the left ventricle PK protein isoform content revealed that PKM2, but not PKM1, protein, was significantly increased in chronically hypoxic hearts, suggesting that sustained *in vivo* hypoxia regulates cardiac PK flux through increased PKM2 protein. This finding extends understanding of the previously reported increased PKM2 expression following acute hypoxia in isolated cardiomyocytes [22], rat H9C2 cells [29] and myocardial infarction [39,41]. In support of its central role in metabolic reprogramming, re-expression of PKM2 has been proposed to regulate the ‘Pasteur effect’ [27,42]. For example, increased PKM2 activity in HeLa and H1299 oncogenic cells has been associated with increased lactate production [19,40]. Conversely, overexpression of PKM2 in C2C12 cells increased glucose consumption without affecting lactate production [20], indicating that PKM2 may play a tissue-specific role in the regulation of energy metabolism.

### Hypoxia up-regulated cardiac PPP flux

Present detailed metabolomic analysis of the heart showed increased R5P content indicative of up-regulated PPP flux, consistent with previous studies [43,44], implying that glycolytically derived carbon backbone was shifted away from upper glycolysis towards PPP, and returned to lower glycolysis via transketolase (TK) and transaldolase (TA) reactions. Although it is incompletely understood how hypoxia remodelled central carbon metabolism in the heart, reduced levels of cardiac NADPH and lactate/pyruvate ratio suggest disrupted redox balance, previously shown to modulate carbon flux in cells exposed to hydrogen peroxide [45]. The fate of accumulated pyruvate in the present study, however, remains unclear. Given the antioxidant properties of pyruvate [46,47], it could be argued, however, that hypoxia-induced cardiac pyruvate accumulation serves as a protective mechanism against oxidative stress to regulate PKM2 activity [2,24,48]. Beyond its role in the control of the glycolytic flux, some [49], but not all [50], authors suggested that PKM2 can also act as a transcriptional factor (protein kinase) to regulate gene expression. However, further studies are necessary to explore the transcriptional role of the PKM2 in the chronically hypoxic hearts.

### Regulation of hypoxia-induced PKM2 expression

Hypoxic regulation of cardiac PKM2 expression is not well understood. Following short-term hypoxia of isolated rat cardiomyocytes, HIF-1α signalling has been shown to regulate PKM2 expression [22]. There are other known regulators of PKM2 expression. Overexpression of CUGBP1 in skeletal muscle has been shown to increase PKM2 protein, whereas Sp3-mediated transcriptional depression has been implicated in the regulation of PK expression during short-term hypoxia in C2C12 cells [20,28]. Our data suggest that *in vivo* sustained hypoxia does not seem to affect HIF-1α signalling, at least in that we find unaltered cardiac PDK1 levels, which is consistent with previous reports on the transient nature of HIF-1α up-regulation during prolonged hypoxic exposure [51–53]. Also, cardiac CUGBP1 levels did not change after *in vivo* hypoxia, suggesting that, while CUGBP1 may acutely regulate skeletal muscle PKM2 expression [20], this mechanism might not operate in chronically hypoxic hearts. Similarly, we also found that our *in vivo* chronic hypoxia does not change the steady-state levels of cardiac Sp3 protein, unlike short-term *in vitro* hypoxic exposure [28]. Therefore, our results seem to indicate that HIF-1α, CUGBP1 or Sp3 signalling were not involved in the increased expression of cardiac PKM2 protein during chronic hypoxia.

## Conclusions

In summary, the findings of the present study indicate that cardiac glycolytic flux during sustained *in vivo* hypoxia appears to be maintained via translational up-regulation of a single rate-limiting enzyme – PKM2. Furthermore, cardiac glycolytic flux during prolonged hypoxia is, at least, partly driven by the up-regulation of the flux through the PKM2. In the light of pre-clinical findings showing increased PKM2 expression in the failing heart, further research is warranted to explore its activators as regulators of cardiac function and metabolism.

## Supplementary Material

Supplementary Table S1

## Data Availability

All data generated or analysed during the present study are included in this published article.

## Abbreviations

CH: chronic hypoxic
DP: developed pressure
FBP: fructose-1,6-bisphosphate
GAPDH: glyceraldehyde-3-phosphate dehydrogenase
HIF: hypoxia-inducible factor
HK: hexokinase
HR: heart rate
KH: Krebs–Henseleit
LC-MS/MS: liquid chromatography tandem mass spectrometry
PEP: phosphoenolpyruvate
PFK: phosphofructokinase
PGK: phosphoglycerate kinase
PK: pyruvate kinase
PKM1: PK muscle isoenzyme M1
PKM2: PK muscle isoenzyme M2
PPP: pentose phosphate pathway
R5P: ribose-5-phosphate
