# Supplementary material for: Potential role for pyruvate kinase M2 in the regulation of murine cardiac glycolytic flux during in vivo chronic hypoxia
Source: Biosci Rep. 2021 Jun 2;41(6):BSR20203170. doi: 10.1042/BSR20203170 (PMC8173528; doi:10.1042/BSR20203170)
Supplement: Supplementary Table S1 [file bsr-2020-3170_supp.pdf]

Supplementary Table 1. LC-MS/MS parameters.

COMPOUND	Parent Ion	Daughter Ion	Collision Energy (eV)
Glucose-6-phosphate	259.0	97.0	15
Fructose-6-phosphate	259.0	97.0	13
Fructose-1,6-bisphosphate	339.0	97.0	19
3-Phosphoglycerate	185.0	97.0	14
Phosphoenolpyruvate	167.0	78.8	11
Pyruvate	87.0	43.1	8
Lactate	89.0	43.3	9
Oxaloactate	131.0	87.0	8
Citrate	191.0	111.0	11
α-ketoglutarate	145.0	101.0	7
Succinate	117.0	73.0	10
Fumarate	115.0	71.0	6
Malate	133.0	115.0	9
Alanine	88.0	88.0	1
6-Phosphogluconate	275.0	97.0	15
Ribose-5-phospate	229.0	97.0	10
NADPH	744.0	664.1	35
